# The Association of Molecular Biomarkers in the Diagnosis of Cervical Pre-Cancer and Cancer and Risk Factors in Senegalese

**DOI:** 10.31557/APJCP.2020.21.11.3221

**Published:** 2020-11

**Authors:** Dominique Diouf, Gora Diop, Cheikh Fall, Souleymane Sarr, Cheikh Ahmed Tidiane Diarra, Aminata Issa Ngom, Sidy Ka, Seynabou Lo, Oumar Faye, Ahmadou Dem

**Affiliations:** 1 *Laboratory of Cytogenetic and Reproductive Biology, Hospital Aristide-Le-Dantec, Pasteur Avenue, Dakar, Senegal. *; 2 *Laboratory of Anatomy and Pathology, Principal Military Hospital of Dakar, Nelson Mandela Avenue, Dakar, Senegal. *; 3 *Cancer Studies and Research Group in Senegal (GERCS), FMPO, Cheikh Anta DIOP University, Cheikh Anta Diop Avenue, Dakar, Senegal. *; 4 *Cheikh Anta DIOP University, Department of Animal Biology, Faculty of Science and Technology, Postulant Unit of Genetic, Genomic and Bioinformatic of Infectious Diseases and Cancer, Cheikh Anta Diop Avenue, Dakar, Senegal. *; 5 *Institut Pasteur Dakar, Pole d’immunophysiopathologie des maladies infectieuses (Pole IMI), Pasteur Avenue, Dakar, Senegal. *; 6 *Institut Pasteur Dakar, Pole de Virologie, PO Box 220. 36, Pasteur Avenue, Dakar, Senegal. *; 7 *Hopital Aristide LeDantec, Service de Cancerologie, Institut-Juliot-Curie, Pasteur Avenue, PO Box 3001, Dakar, Senegal. *

**Keywords:** Biomarkers, cervical cancer, cofactors, human papillomavirus, Senegal

## Abstract

**Background:**

Cervical intraepithelial neoplasia (CIN) grading is subjective and affected by substantial rates of discordance among pathologists. Although recent studies have suggested that p16INK4a may be a useful surrogate biomarker of cervical neoplasia, Ki-67 and human papillomavirus testing have also been shown to be useful in detecting neoplasia. The purpose of this study was to determine the expression of p16INK4a and Ki-67 in cervical neoplasia and its correlations with cofactors.

**Methods:**

The study involved 69 patients with and without cervical neoplasia who underwent colposcopic directed biopsy. On each patient, two samples were taken; the first was used for immunohistochemistry and the second for molecular testing, using HPV16and18 genotyping Real-Time PCR Kit.

**Results:**

The study revealed the expression level of p16INK4a and Ki-67 in a descending order, from invasive squamous cell carcinoma (SCC), CIN2/3, CIN1 and non-dysplastic lesions. Correlations showed an association between the staining of p16NK4a and Ki-67 with the increase of age (OR: 1.79 (95%IC: 0.49 – 6.55), p = 0.037) and marital status (OR: 0.17 (95%IC: 0.04 – 0.68), p = 0.003). We found that the expressions of p16INK4a and Ki-67 were significantly different between invasive SCC vs non-dysplasia (OR: 44.57 (95%IC: 4.91 – 403.91), p<0.0001). The study showed significant correlation between HPV 16and18 infection with p16 INK4a and Ki-67 expression (OR: 0.13 (95%IC: 0.03 – 0.52), p<0.0001). Strong expression of p16INK4a and Ki-67 were observed in invasive squamous cell carcinoma, moderate staining was found in CIN2/3, weak staining in CIN1 and normal histology.

**Conclusion:**

Our findings indicate that p16INK4a and Ki-67 expressions associated strongly with cervical pathology. Therefore, p16/Ki-67 could be considered as a suitable biomarker for cervical cancer screening, particularly in HPV-based screening programs.

## Introduction

With an estimated 570,000 cases and 311,000 deaths worldwide, cervical cancer ranks as the fourth most frequently diagnosed cancer and the fourth leading cause of cancer-related death in women (Bray et al., 2018). In developing countries where access to screening is absent or limited, cervical cancer is much higher than in developed countries where this disease was the seventh leading cause of cancer-related death (Bray et al., 2018). Senegal was 17^th^ of cervical cancer incidence rank worldwide, which is the leading cause of female cancer death with an estimated 1,876 cases diagnosed annually, of which 1,367 cases (72.90%) are fatal (Castellsague and Munoz, 2003; Lee et al., 2006). 

Cervical cancer is in fact rare; only a very small proportion of HPV infected women will develop a high-grade cervical intraepithelial lesions (CIN2+) and even fewer will develop cancer (Wang et al., 2015; Sundström et al., 2013). Cervical cancer is known to develop from precancerous disease, cervical intraepithelial neoplasia (CIN). CIN takes 5 to 15 years to progress to invasive cancer (Castanon et al., 2018; Johnson et al., 2018; Schiffman et al., 2011). 

Pap smear has led to a substantial reduction of cervical cancer incidence in numerous countries. However, Pap cytology has limited reproducibility and a single Pap test has limited sensitivity to detect cervical pre-cancer. HPV DNA testing is highly sensitive and provides a high reassurance of low risk of cervical cancer among women testing negative, permitting safe extension of screening intervals (Finocchario-Kessler et al., 2016; Sundström, 2013). 

The use of prognostic biomarkers is important in order to modulate the management of women who were positive at the first level test, i.e. Pap test or HPV DNA, but also to modulate the intensity of the post-colposcopy follow up. The most promising biomarkers to date are those linked to E6/E7 mRNA and P16NK4a, alone or combined with Ki-67 (Lee et al., 2006 ; Von Knebel, 2002). 

Like the p16INK4a protein, HPV infection induces the release of E2F through the binding of E7 to pRb. The released E2F stimulates the expression of genes, which are involved in G1-S transition (Dyson et al., 1989; Nam et al., 2007). The inactivation of pRb by E7 causes the p16INK4a overexpression because p16INK4a is regulated by negative feedback of pRb (van Zummeren et al., 2018; Walts and Bose, 2009; Nam et al., 2008; Brown et al., 2012).

Ki-67 is a nuclear and nucleolar protein expressed only in active phases and cell cycle (G1, S, G2, and M phases) but not in resting phases (G0 and early G1). Overexpression of Ki-67 correlates with high cellular proliferation. Since HPV infection leads to increased epithelium cell proliferation in infected tissue, increased Ki-67 staining can be an indicator of HPV (Conesa-Zamora et al., 2009a; Bruni et al., 2019). In certain cases, the reactive changes, immature metaplasia or atrophic changes of cervix may show similar morphologic features as intraepithelial lesion or discretion between low-grade lesion and high-grade lesion is not possible by the routine hematoxylin and eosin stain of tissue, the study of these 2 molecular biomarkers may be useful (Conesa-Zamora et al., 2009a; Kava et al., 2015). 

The purpose of this study was first, to analyze expression of p16INK4a and ki-67 in cervical biopsy specimens covering the diagnostic of normal, precancerous, and invasive squamous cell carcinoma. 

Further, we aim to determine the relationship between the severity of cervical histology and the intensity of p16INK4a and ki-67 staining ; then to evaluate the expression of p16INK4a and ki-67 in correlation with biological and socio demographic parameters, and finally to evaluate the correlation of p16INK4a and ki-67 staining with high-risk HPV 16 and18 infection.

## Materials and Methods

*Ethical clearance*

Objectives and benefits were explained clearly using local dialect before inclusion. Protocol has been reviewed according to the rules issued by the National Committee for Ethics for Health Research (CNERS) of Senegal, and in accordance with the procedures established by the University Cheikh Anta Diop of Dakar (UCAD) for the ethical approval of any research involving human participants. Written informed consent was obtained from all participants. Based on these informations, UCAD’s Committee on Research and Ethic (CER) considers that the proposal respects the appropriate ethical standard and approves its execution under “Protocole 0194/2016/CER-UCAD”. 

*Subjects and tissue specimens*

The study involved 69 cervical biopsy specimens from patients who had indication to undergo colposcopy-directed biopsy. 

Patients with invasive carcinoma, dysplasia or normal histology were selected among those who came for routine screening in Gaspard Camara health center and Dantec hospital between January 2017 and December 2018. Invasive squamous cell carcinoma (SCC) were taken in cancer department of Dantec hospital (19 biopsies) among patients who came for chemotherapy/ radiotherapy. On each patient, two fragments of tissue were taken. The first was used for P16INK4a and Ki-67 immunostaining and the second for HPV16/18 genotyping assay using the Bioneer device. 

*Materials*

*HPV 16and18 Detection and genotyping*

The HPV16 and HPV18 genotyping was performed using the molecular AccuPower^®^ HPV 16and18 Real-Time PCR Kit for the simultaneous detection and differentiation of human papillomavirus (HPV) type 16 and 18. Viral DNA was extracted from cervical swab samples or homogenate of biopsy specimens using ExiPrep™ Dx Viral DNA/RNA Kit (Bioneer Corporation is Korea’s leading biotech company) according to the manufacturer recommendation. Briefly, 200 µL of homogeneous solution was transferred to sample tubes and inserted into the ExiPrep™ 16 Dx or 48 Dx instruments for nucleic acid extraction for 90min. A total of 50uL was eluted directly into the PCR tubes contained lyophilised primers/probe as well as internal control and well-sealed before being transferred into the Exicycler™ 96 device for Real-time PCR amplification for 90min. Result analysis was done using the ExiStation™ Manager software.

*Immunohistochemical Staining for p16INK4a and Ki-67 expression*

Formalin-fixed, paraffin-embedded tissue blocks were sliced in thickness of 3µm and the tissue sections were mounted on silanized slides. Immunohistochemical staining was performed through the indirect biotin streptavidin method using the iVIEW™ DAB Detection Kit (Ventana Medical Systems, Tucson, AZ, USA). The sections were deparaffinized in xylene and were sequentially washed twice in 100% alcohol and in 95%, 90%, 80%, and 70% alcohol for two minutes. To increase the antigen detection, the slides were immersed in a citrate acid solution and were heated for 20 minutes in a microwave. The slides were washed with APK Wash Solution (Ventana Medical Systems, Tucson, AZ, USA) and were stained using the automatic immunohistochemical staining equipment, Ventana NexES IHC (Ventana Medical Systems, Tucson, AZ, USA). The p16INK4a and Ki-67 staining was performed with 1:25 diluted Monoclonal Mouse Antibody p16INK4a protein (Diagnostic Bio-System, USA) and 1:50 diluted Monoclonal Mouse Antibody (DAKOCytomation, Benchmark), respectively.

After incubation with antibodies for 32 minutes, the slides were exposed to Diaminobenzidine (DAB) for 4 minutes and were counterstained with Mayer’s Hematoxylene for 4 minutes. DAB and Mayer’s Hematoxylene which were included in iVIEW™ DAB Detection Kit (Ventana Medical Systems, Tucson, AZ, USA) were used for staining. All staining procedures were performed at 37^o^C. Stained slides were dried and were covered with glass cover slides. For a negative control, non-immune mouse serum IgG was used instead of primary antibodies.

*Scoring of p16INK4a*

Diffuse or ‘block’ staining for p16INK4a of the cell cytoplasm or nucleus in squamous epithelium was considered positive. Score 0 (weak expression found in non-dysplasia) is defined as either no p16ink4a positivity or focally scattered positive cells or small cell clusters (i.e., patchy staining). Score 1 (weak expression found in CIN1) is defined as low intensity, diffuse positivity restricted to the lower one-third part of the epithelium. Score 2 (medium expression found in CIN2/3) is defined as continuous positivity in the lower two-thirds of the epithelium. Score 3 (strong expression found in SCC carcinoma) is defined as positive cells involving the full thickness of the epithelium (i.e., diffuse full thickness staining) (van Zummeren et al., 2018).

*Scoring of Ki-67*

Nuclear Ki-67 staining in cells of the squamous epithelium was scored positive. Score 0 (weak expression found in non-dysplasia) is a normal staining pattern (i.e., staining of nuclei in the basal layer). Score 1 (weak expression and found in CIN1) is defined as positive nuclei predominantly found in the lower one-third of the epithelium. Score 2 (medium expression and found in CIN2/3) is defined as positive nuclei predominantly found in the lower two-thirds of the epithelium. Score 3 (strong expression found in SCC carcinoma) is defined as positive nuclei in more than two-thirds of the epithelium (van Zummeren et al., 2018).

*Statistical analysis*

The analyzed data were performed with IBM SPSS Statistics version 24.0. For the analysis of descriptive statistics, frequency and percentage were used to describe the qualitative variable and dispersion and trend indicators (mean, mode, median, maximum and minimum, standard deviation) were used for continuous variables. Expression of p16INK4a and Ki-67 staining in association with the results of histopathology, HPV testing and socio-demographic parameters was compared using the chi-square test. A value p <0.05 was considered as statistical significance. 

## Results

*p16INK4a and Ki-67 Expression with the sociodemographic characteristics (* Table 1 *)*

Fourteen patients with CIN1 (20%), 3 patients with CIN2 (4.34%), 3 patients with CIN3 (4.34%), 19 patients with invasive SCC (27.53%) and 30 patients with normal histology (43.47%) were included in this study. The Table 1 summarized the sociodemographic characteristics of included subjects. The p16INK4a staining and the Ki-67 were performed in 46 patients. In addition, analysis showed a signification between p16INK4a and Ki-67 expressions with age OR:1.79 (95%IC: 0.49 – 6.55, p=0.037), and marital status ( OR: 0.17 (95%IC: 0.04 – 0.68, p = 0.003). The association with parity, education and the first marriage and contraceptive use were less statically significant, as shown in Table 1. 

*p16INK4a and Ki-67 Expression with the cervical histopathology*

**Figure 1:**
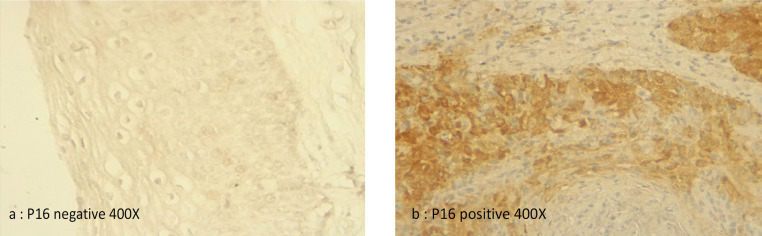
Representative picture of immunohistochemical analysis of p16INK4a (p16) Expression in Cervical Tissue (400X)

**Figure 2:**
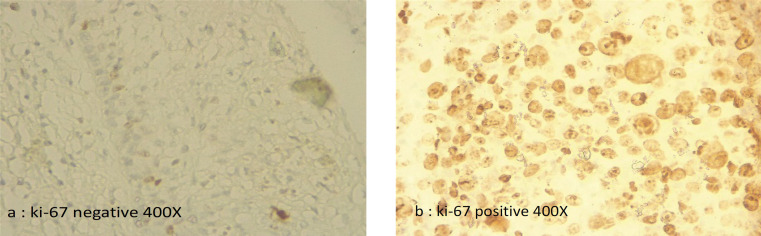
Representative picture of Immunohistochemical Analysis of ki-67 Expression in Cervical Tissue (400X)

The rates of p16INK4a and Ki-67 expression were in descending order from invasive SCC to CIN2/3, CIN1 and non-dysplastic lesions. Therefore, the expression of p16INK4a (Figure 1) and Ki-67 (Figure 2) were 100% in all squamous cell carcinoma and CIN2/3, for CIN1 the expression of p16INK4a and Ki-67 was 43%, and for non-dysplasia the expression of p16INK4a and Ki-67 was 14%. We found that the expressions of p16INK4a and Ki-67 were significantly different between the cervical histopathology (Non-dysplasia, CIN1, CIN2/CIN3, invasive SCC) (OR 44.5 (95%CI: 4.91 – 403.91, p<0.000) (Table 2).

*Intensity of p16INK4a and ki-67 immunohistochemical staining related to cervical histopathology*

Strong expression of p16INK4a and Ki-67 were observed in all 19 invasive SCC, moderate expression was found in CIN2/3 and weak expression was shown by CIN1 (Table 4). Altogether, the grade of histological abnormality is correlated with the level of expression of p16INK4a and Ki-67 staining, and as abnormality is higher, stronger p16 and Ki-67 expression were observed. Positive rates of Ki-67 and P16INK4a expression in HSIL and SCC groups were significantly higher than those in LSIL and non-neoplasia. 

*p16INK4a and Ki-67 expression with the HR-HPV status (* Table 3 *)*

HPV test was performed in 38 patients in which 3 patients were infected by HPV-18 and 32 patients by HPV16. Interestingly, 2 patients were co-infected by HPV16/18. The study showed significant correlation between p16INK4a/Ki-67 expression and CIN2/3 and invasive SCC (OR 0.13 (CI 0.03 – 0.52) p: <0.01) and HR-HPV (OR 0.13 (CI 0.03 – 0.52) p <0.01)

Among patients which showed p16INK4a/Ki-67 expression, 1 patient with non-dysplasia was infected by HR-HPV, 4 patients with CIN1 were infected by HR-HPV and all CIN2/3 and invasive SCC were infected by HR-HPV (Table 3). 

## Discussion

To our knowledge, this is the first Senegalese study to evaluate the interest of p16INK4a and Ki-67 in histological samples. This study focused first on the correlation between p16INK4a and Ki-67 with the histological abnormalities, then the association between the degree of abnormality and the expression level of p16INK4a and ki-67 and last, the correlation between the HPV16 and18 infection and, the sociodemographic characteristics. The study, which included 69 patients, showed an association between the expression of p16INK4a and Ki-67 with patient age and marital status. These two cofactors are known to be associated with the increase of cervical pathology which explain their correlation with the expression ofp16INK4a and Ki-67. These results agreed with those found by Yetimalar (2012), concerning the patient age, however the marital status was not consistent for those studies. Contrary to Yu (2016) report highlighting the association of parity, education, menopause with p16INK4a and Ki-67 expressions.

We evaluated p16INK4a and Ki-67 expression in different histological categories, and found that positivity of p16INK4a and Ki-67 increased significantly with disease severity. Indeed, p16INK4a and Ki-67 expressed in all the invasive SCC and CIN2/3 (100%), as well as 42.85% and 50% in CIN1 for p16INK4a and Ki-67 respectively, and 14.2% for non-dysplasia. These results were consistent with the study of Wentzensen (2015). The overexpression of p16INK4a in cervical dysplasia was reported to be associated with the transforming activity of the E7 oncoprotein of HR-HPV types through inactivation of the tumor-suppressor function of the retinoblastoma protein (pRb) (Thomsen et al., 2015). High correlation was also found between CIN and p16INK4a/Ki-67 expression in many previous studies evaluating immunohistochemical expression of biomarkers in cervical intraepithelial lesions as an adjunct for a diagnosis of cervical squamous intraepithelial lesion and invasive SCC. Previous studies found p16INK4a expression in 91% of invasive, 78% CIN2/3, only 10% in CIN1 and 9% in non-dysplasia (Ferlay et al., 2018; Nam et al., 2006). Other studies reported p16INK4a in 80% to 100% in invasive carcinoma, 45% to 100% in CIN2/3, and 0% to 15% in non-dysplasia (Bray et al., 2018; Kim et al., 2015; Volgareva et al., 2004; Sarma et al., 2017; Wentzensen et al., 2019). Immunopositivity for Ki-67, a biomarker for cell proliferation, increase linearly as the CIN grade is higher (Lee et al., 2006). Kanthiya (2016) found Ki-67 expression in 100% of invasive cancer, 75% in CIN2/3, only 22% in CIN1 and 11% in non-dysplasia. Other studies found Ki-67 in 90% to 100% in invasive carcinoma, 20% to 70% in CIN2/3, 70% to 90% in CIN1, and 0% to 20% in non-dysplasia (Castellsague and Munoz, 2003). These results are concordant with our findings. 

The low sensitivity of Pap smears and low specificity of HPV testing by HybridCapture2 are other factors which signifies the importance of the biomarkers. The role of HRHPV in the causation of cervical dysplasias and neoplasia further helps to identify the utility of these markers, especially p16INK4a as its high expression is known to correlate with the prevalence of HRHPV types (Hebbar and Murthy, 2017 ; Jiang et al., 2020).

Regarding the association between HR-HPV and p16INK4a as well as Ki-67 expression, most researchers believe that p16INK4a is a surrogate marker for CIN induced by HR-HPV and that HR-HPV negative status accompanied by p16INK4a positivity is often regarded as a false negative (Zong et al., 2015).

The coexpression of p16INK4a and Ki-67 was developed as an auxiliary marker of cervical pre-cancers (Kanthiya et al., 2016; Sundström et al., 2013) but a series of studies reported that an increased risk of high grade CIN or cervical cancer is associated with high HPV DNA loads, suggesting that HPV is a marker to predict cervical neoplasia (Lim et al., 2016 ; Zong et al., 2015 ; Nam et al., 2008).

An association between p16INK4a/Ki-67 expression and HPV16and18 infection was found in our study with a significant higher positivity rate of p16INK4a and Ki-67 immunohistostaining in HPV16and18 infection group compared to women without HPV16and18 infection (OR: 0.13, 95%IC: 0.03 – 0.52, p<0.0001). These findings are consistent with those found by Lim et al., (2016), Li et al., (2019); Tay et al., (2017) and Ebisch et al., (2017).

But the studies contrast with analysis made by Dyson et al., (1989) which showed poor correlation between p16INK4a as well as Ki-67 positive ratio and HPV infected status. The difference of these findings, with our results could be linked to the fact that samples used by Dyson et al., (1989), Sarma et al., (2017) were only CIN1.

We evaluated p16INK4a and Ki-67 expression in different histological categories, and found that the expression level of p16INK4a and Ki-67 increased significantly with disease severity. Indeed invasive carcinoma showed high-level expression of p16INK4a and Ki-67, the high grade (CIN2/3) showed 50% of medium expression for p16INK4a while Ki-67 showed 66% medium expression. The level expression for CIN1 was weak. These findings were concordant with previous studies, which found high-level expression status of p16INK4a in high squamous intraepithelial lesions (HSIL) and weak level expression in low squamous intraepithelial lesion (LSIL) (Li et al., 2019; Marjolein et al., 2018; Schiffman et al., 2007). The findings are in accordance with the study by Lesnikova (2009) who also found progressive overexpression of p16INK4a from CIN1 (43.3%) to CIN 3 (94%).

Our results suggest that p16/Ki-67 is a suitable biomarker for cervical cancer screening, particularly in HPV-based screening programs. This should encourage efforts of the government to implement screening programs and possibly as a surrogate marker for HPV infection and emphasizes towards the use of vaccination against HPV infection. In addition to the current government efforts to implement nationwide screening programs, information about signs and symptoms of the disease should be spread.

This study has some weakness such as the small number of patients included in this study. Moreover, the genotyping test used in the study targeted only HPV16 and 18, which may constitute a bias, as some patients may be infected with other high-risk HPV types.

In conclusion, p16INK4a and Ki-67 expression were directly associated with the severity of cervical lesions. The highest expression of both markers was found in invasive SCC and CIN2/3 and lower in descending order for CIN1. The two markers are efficient in advancing the diagnostic accuracy of cervical biopsies in cases of high risk HPV (HPV16and18). However, pathologists should be aware that unusual immunostaining results in HPV-negative patients, such as negative p16INK4a staining in HSIL, may imply factors other than high risk HPV infection. Moreover, an analysis implicating larger sampling size are needed to confirm obtained results

**Table 1:** Correlation between Socio-Demographic Characteristics with p16INK4a and Ki-67 Expression status (N = 46)

Characteristics	p16INK4a/Ki-67		Multivariate analysis,	p-value
	Negative	Positive	OR (95%IC)	
Age (yr)			1.79 (0.49 – 6.55)	p= 0.037
≤ 45	7 (15.22)	13 (28.26)		
≥ 45	7 (15.22)	19 (41.30)		
Education			5.94 (1.47 – 23.97)	p=0.051
High Level	9 (19.57)	6 (13.04)		
Low Level	5 (10.87)	26 (56.52)		
Parity			2.39 (0.61 – 9.32)	P= 0.079
0 – 5	10 (21.74)	15 (32.61)		
>5	4 (8.70)	17 (36.95)		
Age at first marriage			0.40 (0.08 – 1.97)	P= 0.527
<18	7 (15.21)	12 (26.09)		
≥18	12 (26.09)	15 (32.61)		
Contraceptive use			0.80 (0.16 – 3.83)	P= 0.055
hormonal	11 (23.91)	26 (56.52)		
Non-hormonal	0 (0)	2 (4.35)		
Non-users	3 (6.52)	4 (8.70)		
Marital status			0.17 (0.04 – 0.68)	P= 0.003
Monogamous husband	7 (15.22)	8 (17.39)		
Polygamous husband	4 (8.70)	22 (47.82)		
Not married	3 (6.52)	2 (4.35)		

Data shown are number (%) not otherwise specified. The result is significant at p < 0.05

**Table 2:** Expression of p16INK4a and Ki-67 in Relation to Histological Diagnosis N = 46

Histological diagnosis	P16/Ki-67 Positive n (%)	P16/Ki-67 Negative n (%)	Multivariate analysis OR (95%IC)	P-value
Non-dysplasia	1 (14%)	6 (86%)	44.57 (4.91 – 403.91)	0
CIN1	6 (43%)	8 (57%)	44.57 (4.91 – 403.91)	0
CIN2/CIN3	6 (100%)	0 (0%)	44.57 (4.91 – 403.91)	0
Invasive SCC	19 (100%)	0 (0%)	44.57 (4.91 – 403.91)	0

CIN, Cervical Intraepithelial Neoplasia; SCC, Squamous Cell Carcinoma CI Confidence interval; OR, odds ratio; The result is significant at p < 0.05.

**Table 3:** Expression of p16INK4a and Ki-67 in Relation to hr-HPV Status (N = 38)

Histological Diagnosis	p16 and Ki-67 positivity		OR (95%IC)	P-value
	HR-HPV Negative	HR-HPV Positive		
Non-dysplasia	6 (85%)	1 (15%)	0.13 (0.03 – 0.52)	0
CIN1	2 (33%)	4 (67%)	0.13 (0.03 – 0.52)	0.002
CIN2/CIN3	0 (0%)	6 (100%)	0.13 (0.03 – 0.52)	0.01
Invasive SCC	0 (0%)	19 (100%)	0.13 (0.03 – 0.52)	0

CIN, Cervical Intraepithelial Neoplasia; HR-HPV, High Risk Human Papilloma Virus; SCC, Squamous Cell Carcinoma CI Confidence interval, OR, odds ratio; The result is significant at p < 0.05

**Table 4:** Intensity of p16INK4a and ki-67 Immunohistochemical Staining Related to Grade of CIN

Histological Diagnosis	p16INK4a Score				Ki-67 Score			
	0	1	2	3	0	1	2	3
Non-dysplasia	6 (85.7)	1 (14.3)	0 (0)	0 (0)	6 (85.7)	1 (14.3)	0 (0)	0 (0)
CIN1	8 (57.1)	4 (28.6)	214.3)	0 (0)	7 (50)	5 (35.7)	2 (14.3)	0 (0)
CIN2/CIN3	0 (0)	1 (14.3)	3 (42.85	3 (42.85	0 (0)	0 (0)	4 (66.7)	2 (33.3)
Invasive SCC	0 (0)	0 (0)	0 (0)	19 (100)	0 (0)	0 (0)	0 (0)	19 (0)

0, (no staining); 1, (rare singly dispersed cells staining); 2, (patchy but strong staining, often not continuous from basement membrane); 3, (strong and diffuse staining from basement membrane and extending upward in proportion to lesion grade.
